# Influence of physical exercise on microRNAs in skeletal muscle regeneration, aging and diseases

**DOI:** 10.18632/oncotarget.24991

**Published:** 2018-03-30

**Authors:** Simona Ultimo, Giorgio Zauli, Alberto M. Martelli, Marco Vitale, James A. McCubrey, Silvano Capitani, Luca M. Neri

**Affiliations:** ^1^ Department of Morphology, Surgery and Experimental Medicine, University of Ferrara, Ferrara, Italy; ^2^ Department of Biomedical and Neuromotor Sciences, University of Bologna, Bologna, Italy; ^3^ Department of Medicine and Surgery, University of Parma, Parma, Italy; ^4^ CoreLab, Azienda Ospedaliero-Universitaria di Parma, Parma, Italy; ^5^ Department of Microbiology and Immunology, Brody School of Medicine, East Carolina University, Greenville, USA

**Keywords:** skeletal muscle, regeneration, aging, physical activity, miRNAs

## Abstract

Skeletal muscle is a dynamic tissue with remarkable plasticity and its growth and regeneration are highly organized, with the activation of specific transcription factors, proliferative pathways and cytokines. The decline of skeletal muscle tissue with age, is one of the most important causes of functional loss of independence in older adults. Maintaining skeletal muscle function throughout the lifespan is a prerequisite for good health and independent living.

Physical activity represents one of the most effective preventive agents for muscle decay in aging.

Several studies have underlined the importance of microRNAs (miRNAs) in the control of myogenesis and of skeletal muscle regeneration and function.

In this review, we reported an overview and recent advances about the role of miRNAs expressed in the skeletal muscle, miRNAs regulation by exercise in skeletal muscle, the consequences of different physical exercise training modalities in the skeletal muscle miRNA profile, their regulation under pathological conditions and the role of miRNAs in age-related muscle wasting.

Specific miRNAs appear to be involved in response to different types of exercise and therefore to play an important role in muscle fiber identity and myofiber gene expression in adults and elder population.

Understanding the roles and regulation of skeletal muscle miRNAs during muscle regeneration may result in new therapeutic approaches in aging or diseases with impaired muscle function or re-growth.

## INTRODUCTION

Skeletal muscle upholsters the important role of supporting skeletal structure and movement and it is also involved in glycogen synthesis and amino-acid deposits [1, 2]. A general physical performance is improved by exercise that induces molecular and cellular adaptations [2]. Metabolism of muscle and its homeostasis are due to protein synthesis/degradation and activity of muscle stem cells, whereas the synthesis and degradation of total muscle protein is due principally to nutrition and physical activity seems to act on both protein synthesis/degradation and on satellite cells [2].

At the entire muscle level, hypertrophy is the major consequence of physical exercise that can be produced both by new satellite cell fusion or by amplified protein synthesis [3].

Likely, satellite cells are stimulated when an acute stimulus occurs (i.e. strong exercise with eccentric contractions, that is a motion of an active muscle while it is lengthening under load) and leads to hypertrophy enhancing the number of myonuclei and their fiber size, thus imitating the regeneration pathway in response to injury [4]. Nevertheless, moderate physical activity does not stimulate satellite cells and functional over-load leads to a muscle growth solely through protein synthesis [2, 5].

Physical activity is a term used to describe any physical training developed by skeletal muscles that requires energy cost, which may be unstructured and everyday life motion. The term exercise includes prearranged, deliberate and repetitive motion and ordinary sports and agonistic sports [6].

In chronic degenerative diseases, physical activity is one of the most efficient protective agents and it has been suggested as effective therapy for chronic diseases such as for example osteoarthritis and claudication [7].

Actually, both at genomic and post-genomic levels, a regular physical activity exerts a relevant effect on different parameters and biological pathways [8].

microRNAs (miRNAs) are a class of small non-coding RNAs (around 21–25 nucleotides) that play a role in modulation of gene expression at the post-transcriptional level by inhibiting translation or leading to RNA degradation [2, 9]. In health prevention there is an increasing attention to know more markedly the processes involved in stimulation and inhibition of miRNAs expression. Several miRNAs are essential as mediators of processes associated with exercise training adaptation including cardiac and skeletal muscle hypertrophy and regeneration in adulthood and elder population. Recently it has been shown the effects of regular sport exercise on miRNAs regulation. In addition, miRNAs seem to play important roles in the acute and chronic resistance training (RT), endurance training (ET), in athletes, in animal models, in patients and in the general population [10] and regulation of miRNAs, controlled by physical activity in human skeletal muscle, depends on variety, intensity and duration of the training.

Aging is associated with alterations in skeletal muscle size, structure and function, that may lead to muscle atrophy with increased morbidity and mortality.

During tissue aging, it is well known that both stem cell number and function are reduced [11].

Muscle growth and increased strength is the result of progressive resistive training in older individuals, if the training stimulus is of an adequate intensity and duration. Endurance physical activity in aged, where the stimulus is repeatedly enhanced, evokes a development of muscle capillaries, enhances oxidative enzyme activity, and induces important amelioration of maximal oxygen consumption (VO_2_ max) [12].

Despite a quite large and increased number of studies on miRNAs in skeletal muscle and aging during physical exercise, an analysis that summarized these results has not been already described.

With this review we aim to overview how acute or chronic physical exercise influences miRNA expression and whether the variability of muscle hypertrophy in response to exercise may be attributed to differential miRNA regulation in the skeletal muscle. We describe how miRNAs are differentially expressed in elder age at the baseline condition and the relevance of physical activity.

## SKELETAL MUSCLE REGENERATION AND PHYSICAL EXERCISE

### Aspects of myogenesis

A complex and multi-stage process involving many regulators is named myogenesis. The myogenic progenitor cells confer to proliferating cells commitment to the myogenic lineage (myoblasts) [13]. The process of myogenesis is determined by the myogenic regulatory factors (MRFs) that include four key transcription factors: Myf5, MyoD, Myogenin (MyoG) and Myf4 regulating the differentiation of muscle cells. The determinants of myogenesis are Myf5 and MyoD whereas MyoG and Myf4 are greatly expressed during the terminal differentiation and they drive myoblasts fusion forming myotubes [13].

**Figure 1:**
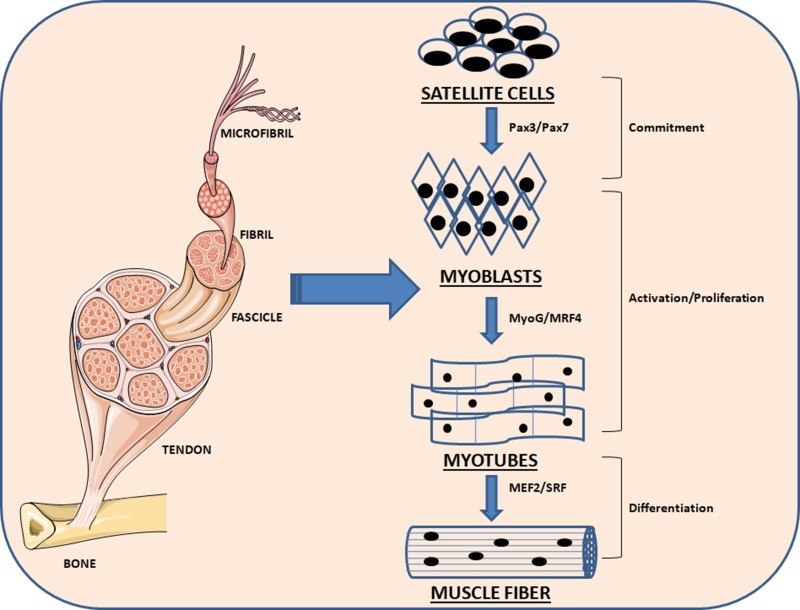
Schematic representation of the myogenesis process Quiescent skeletal muscle satellite cell can become activated following stimuli. The skeletal myoblasts, express transcriptions factors Pax7 and Pax3, as well as the myogenic regulatory factors Myf5 and MyoD. Once committed to differentiation, myoblasts arrest cycling and loose expression of Pax7, Pax3 and Myf5. MRF4 is further required for hypertrophy of the new fibers.

Paired-domain- and homeobox-containing proteins are upstream regulators of early MRFs, including Pax3 and Pax7, which are in action in embryogenesis. When myoblasts move, MyoG and MRF4 are expressed and drive myoblasts to differentiate to myotubes. They operate in concert with other factors following the terminal differentiation into myotubes, including myocyte enhancer factor 2 (MEF2) [14, 15] (Figure 1). Mesoderm-derived structures create the first muscle fibers of the body proper during embryonic myogenesis and consequently waves of additional fibers are created along these template fibers [16, 17].

As described below these transcription factors and regulatory proteins are again involved during muscle regeneration processes.

### The muscle regeneration process and skeletal muscle stem cells

During embryogenesis muscle regeneration after damage or physical training has analogies to muscle development [18].

Skeletal muscle restore is a very synchronized mechanism including the stimulation of different molecular and cellular responses. In addition the coordination among inflammation and regeneration is critical for the positive result of the repair mechanism following muscle injury [19]. Muscle tissue restore can be described as a mechanism following injury consisting of two interdependent phases, degeneration and regeneration, whose successful outcome in muscle repair appears to be affected by the level of the injury and the interactions among muscle and infiltrating inflammatory cells.

A balance among pro-inflammatory and anti-inflammatory factors is correlated to muscle regeneration process that decides whether the injury will be resolved with muscle fiber change and regeneration of a functional contractile apparatus, or with scar formation [18]. The phases of the recovery mechanism are analogous in various causes of injury, and the kinetics and amplitude of each phase may depend on the kind of muscle injuried and the area of injury [18].

The main stem cell pool of adult skeletal muscle are represented by satellite cells [20]. These cells, which were first described by Mauro (1961) [21], represent undifferentiated myogenic precursor cells that lie between the external lamina and sarcolemma of skeletal muscle fibers. Under normal conditions, satellite cells are quiescent, but with appropriate environmental signals they become activated and enter again into the cell cycle to either create novel muscle fibers or supply novel myonuclei to the parent fiber [22, 23]. Studies about genetic ablation and transplantation taken jointly confirmed that Pax7+ satellite cells are required for adult muscle restore [24–26], in response to muscle injury. Satellite cells transition from their normally quiescent state to initiate the cell cycle, expand and differentiate (exiting again from the cell cycle) thus forming novel muscle fibers and regenerating the damaged muscle tissue [27].

An increase in the number of satellite cells is necessary for full skeletal muscle growth and hypertrophy, therefore, the majority of the regeneration process is an orderly sequence of activation, proliferation and differentiation of satellite cells.

Quiescent satellite cells are present throughout the muscle, but the distribution of satellite cells has been shown to vary between different individuals and between different muscle groups and muscle fiber types [28]. Muscles containing mainly type I fibers are very resistant to fatigue and capable of producing repeated low-level contractions (e.g. soleus muscle [29]), type II fibers, known also as fast glycolytic fibers, produce instead fast and strong muscle contractions (e.g. extraocular eye muscle, arm muscles) [30]. The population of satellite cell is not constant: it has been shown to increase after low-frequency exercise and in neuromuscular diseases such as Duchenne muscular dystrophy and neurogenic atrophy, while it has been reported to decrease in olderly men and women compared with a younger population [31]. The signaling pathways for satellite cell activation are not fully understood, but several mediators such as growth factors, nitric oxide (NO), mechanical stimulation and physiological stimuli induced by exercise have been suggested. Identification of satellite cells has commonly been done using electron microscopy and immunohistochemical identification, identifying a cell-surface, membrane-bound neural cell adhesion glycoprotein (N-CAM) [32].

## THE RESPONSE OF SKELETAL MUSCLE REGENERATION TO DIFFERENT PHYSICAL TRAININGS: RESISTANCE AND ENDURANCE

A series of changes in the molecular and structural properties of skeletal muscle are promoted by physical activity, including changes in mitochondrial biogenesis and metabolism, enhanced muscle vascularization and the modulation of myofibrillar content [33, 34]. These alterations are often related to the amelioration of whole-body aerobic ability [35] and although its a greatly adaptable ability, the skeletal muscle could react in different ways to resistance and endurance physical training [34].

Resistance training (RT) is defined as any physical activity that lead to muscle contraction against an external resistance with the expectation of boost in strength, tone and mass. As external resistance, objects can be used such as dumbbells, rubber exercise tubing, your own body weight, bricks, bottles of water, or any other object that leads to muscle contraction to win an external resistance including gravity force [36]. Usually, resistance exercises happen in short bouts with rest periods between each set and are anaerobic.

Endurance training (ET) is the act of exercising to increase endurance, in other words, the ability to keep doing something difficult, unpleasant, or painful for a long time. The term endurance training generally refers to training the aerobic system as opposed to the anaerobic system [37]. Incresed capillarization, amelioration in energy metabolism, mitochondrial biogenesis and the transformation of fast-to-slow fiber type can be caused by ET, whereas RT elicits to the biosynthesis of contractile and structural proteins, leading to muscle hypertrophy and increased generation of contraction force [34].

Resistance and endurance exercise training enhances the satellite cell pool [38]. It was also demonstrated that an increase in the satellite cell pool of skeletal muscle, following ET, depends on the intensity rather than duration of exercise [38]. However, it is not fully clear which factors could induce increase in the proliferative potential of satellite cells by physical training. Skeletal muscle has been acknowledged as a cytokine producing organ during muscular exercise, the so called myokines. To date, the list of identified myokines includes IL-6, IL-7, IL-8, IL-15, leukemia inhibitory factor (LIF), Irisin [39], fibroblast growth factor 21 (FGF 21) and brain-derived neurotrophic factor (BDNF) [40, 41]. Some myokines have the potential to modulate satellite cell proliferation. Previous research showed that dramatic increase in plasma IL-6 and LIF were observed after endurance exercise [42]. Kurosaka *et al* (2012) [38] confirmed that IL-6 may induce a dose-dependent increase of satellite cells through activation of janus kinase (JAK)/signal transducer and activation of transcription 3 (STAT3)/Cyclin D1 pathway that promotes cell cycle activation.

RT exercises increase the cross sectional area (CSA) of the whole muscle together with individual muscle fibers myofibrillar dimension and quantity [43]. The hypertrophy response to RT is associated with the stimulation of early stage satellite cells and may also promote other changes, such as hyperplasia, modifications in muscle fine structure, in myofilament density and in the connective tissue structure [43]. Structural transformations in skeletal muscle during RT are fiber specific: fast-twitch (FT) fibers are more sensitive to injury than slow-twitch (ST) ones [44]. Damage caused by RT in skeletal muscle has been clarified to be also a stimulus for muscle regeneration since it promotes signaling events arised from fiber mechanical deformation, immune/inflammatory responses and hormones production [44]. The synthesis rate of myofibrill is enhanced by RT but not by sarcoplasmic proteins [45].

An age consequence is a slower recovery from RT damaging, whereas recovery from small damaging metabolic fatigue did not report age-related difference [46]. RT increased the level of insulin-like growth factor 1 (IGF-1) and of mechano growth factor (MGF) in skeletal muscle and faster recovery of muscle tissue is supported by both these elements [47].

ET exercises is responsible of most modifications in muscle fibers type I and IIA. The day after ET, important injurious modifications can be observed in these fiber myofibrils. This injury involves the decay of myosin and actin filaments and the regularity of Z-line in some sarcomeres. Myosin filaments were absent after ET in some A-discs and the injurious stress may influence the whole sarcomere. In response to ET 5′ adenosine monophosphate protein kinase (AMPK) is activated and is associated to skeletal muscle metabolic adaptation. AMPK function involves glucose transport, glycogen metabolism, fatty acid oxidation and structural muscle genes transcriptional regulation [48]. AMPK α1 isoform regulates skeletal muscle growth and α2 isoform controls its metabolic adaptation [49].

Protein change in skeletal muscle is quite slow. Myosin heavy chain (MyHC) and myosin light chain (MyLC) isoforms change rate provides a process by which the kind and quantity of protein is modified in agreement with the necessities of the contractile machinery during adaptation to ET. ET increase the satellite cell number mainly under the basal lamina of type I and IIA fibers and increase their regeneration capacity [50].

## MIRNA FUNCTIONS IN SKELETAL MUSCLE REGENERATION

To identify the function and modulation of skeletal muscle miRNAs during different phases of muscle regeneration, in healthy and pathological status, will increase our knowledge of skeletal muscle biology significantly and may result in novel treatments in aging and pathologies related with damaged muscle development, regeneration or activity [51].

miRNAs provide a key and powerful tool in gene regulation in several cellular roles such as progression, differentiation, growth and metabolism. Up to date around 2200 miRNA genes have been described in the mammalian genome [52, 53].

**Figure 2:**
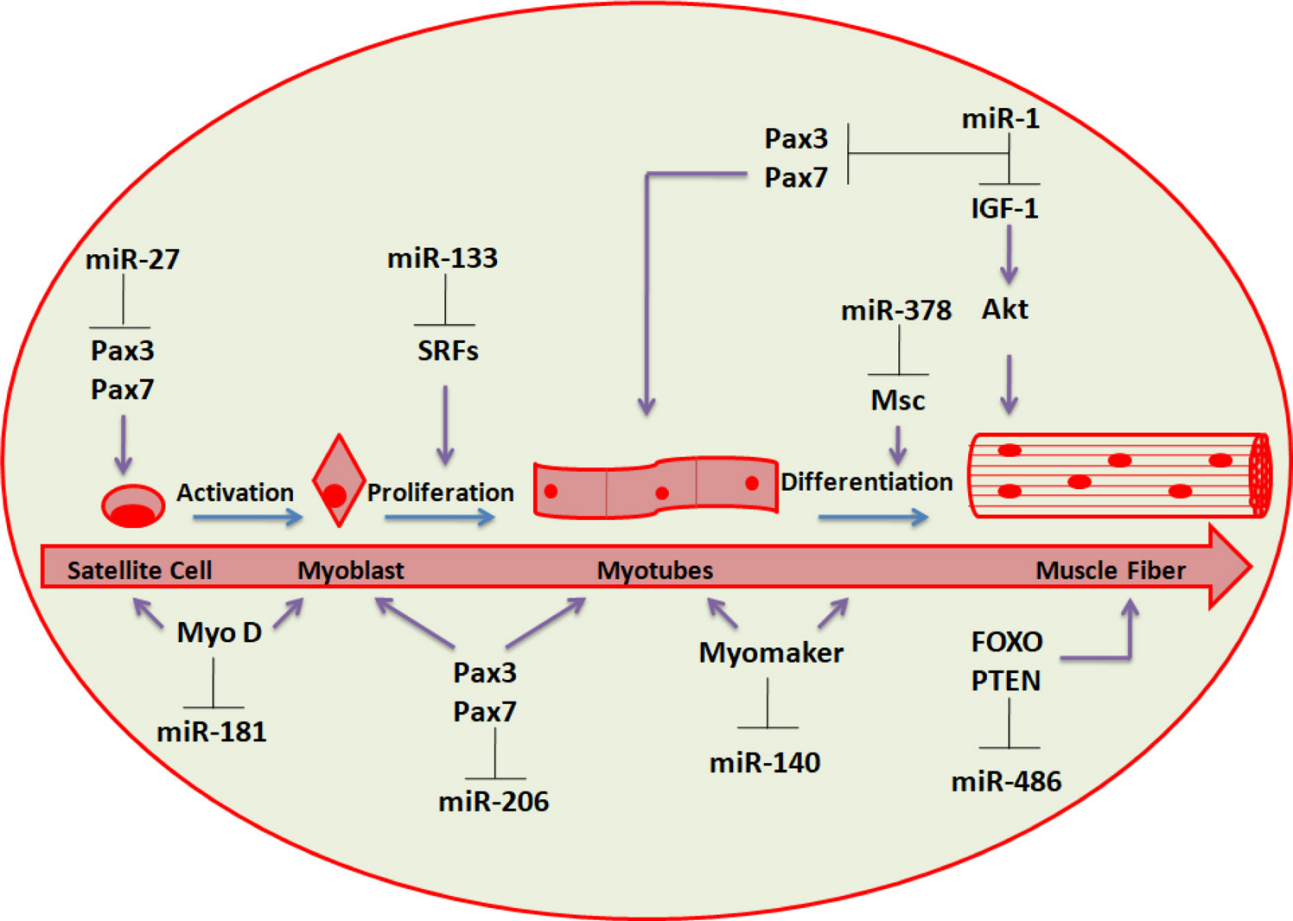
miRNAs involved in skeletal muscle regeneration Schematic representation of the differentiation stages leading from progenitor muscle cells to terminally differentiated fibers. The most relevant regulatory circuits between miRNAs and protein factors are shown.

Several miRNAs, greatly enriched in cardiac and/or skeletal muscle (named myomiRs), has been determined and involve miR-1, miR-133a, miR-133b, miR-206, miR-208, miR-208b, miR-486 and miR-499 (Figure 2) [54–57].

Many of these miRNAs (i.e. miR-1-1/133a-2, miR-1-2/133a-1 and miR-206/133b) are distributed under bicistronic clusters on the same chromosome and are transcribed together [58].

MyomiRs regulation is under the control of MRFs, including MyoD and MyoG [59, 60] as well as by MEF2 [61], SRF [62] and myocardin-related transcription factor-A (MRTF-A) [56]. MyomiRs affect several facets of muscle growth and role, regulating key genes controlling myogenesis [59, 62]. Despite miR-1 and miR-133 tissue-specific expression is driven by MyoD and SRF, miR-133 inhibits SRF expression. In this way, these results recognize a negative regulatory loop in which miRNAs take part to cellular proliferation and differentiation control. Myogenesis is promoted by miR-1 targeting histone deacetylase 4 (HDAC4), a transcriptional repressor of muscle gene expression (Table 1), whereas myoblast proliferation is increased by miR-133 inhibiting SRF [62]. Aberrant regulation of some of these muscle-enriched miRNAs could disrupt intracellular signaling networks [56, 63] which may result in pathological conditions (e.g. muscular dystrophies) [64].

**Table 1:** Summarized list of the most studied miRNAs with their target genes and the type of exercise involved

miRNA	Main target	Type of exercise involved	References
miR-1	HDAC4; Pax3/Pax7; Cyclin D1.	Acute and Chronic Endurance Training	van Rooij E. *et al*.,2009; Margolis LM. *et al*. 2017.
miR-21	PTEN, SPRY-1, ERK/MAP	Acute and Chronic Endurance Training	Iwasaki H. *et al*., 2015; Shen L. *et al*., 2016.
miR-126	RAAS, VCAM-1	Acute and Chronic Endurance Training	Kim E. *et al*. 2012; Shen L. *et al*., 2016.
miR-133a/miR-133b	CALM1, SRF, PTBP2, SP1.	Acute resistance exercise Chronic training, Chronic resistance exercise	Masi LN. *et al*., 2016; Margolis LM. *et al*., 2017.
miR-181a	SIRT1, PTEN, NFATC1.	Acute and Chronic Endurance Training	Silva GJJ. *et al*., 2017; Margolis LM. *et al*., 2017.
miR-206	Pax3/Pax7, BDNF, HDAC4.	Acute and Chronic Endurance Training	Nielsen S. *et al*., 2010; Nielsen S. *et al*., 2014.

Satellite cells and adult muscle stem cells show various sets of miRNAs during quiescence or activation, contributing to muscle regeneration, suggesting miRNA roles in controlling satellite cell homeostasis [65]. In addition, during either physical activity and aging, the expression profile of miRNAs in satellite cells and muscle is modified [66]. The generation of novel muscle fibers and the restore of damaged myofibers (a process that lead to formation of muscle tissue) need the differentiation of myogenic progenitors or satellite cells respectively and many miRNAs have been demonstrated to control muscle regeneration in adulthood, involving miR-1, miR-206, miR-27, miR-378 and miR-181 (Figure 2) [66, 67].

The Pax3 expression is downregulated when satellite cells/myogenic progenitors are activated. If MyoD expression is increased, the Pax3/Pax7 expression downregulation is partially mediated by miR-1 and miR-206, warranting total inhibition of Pax genes to achieve synchronisation of myogenesis regeneration (Table 1) [68]. Moreover, miR-206 and miR-486 have been demonstrated to cause myoblast differentiation by downregulating Pax7 expression and Pax3 expression is inhibited by miR-27 in satellite cells during regeneration process [66, 69, 70].

MyoD activation contributes to the upregulation of several miRNAs expression during myogenesis, including miR-378, an inhibitor of mesenchymal stem cell (Msc) proliferation [71]. miR-378 may strengthen the formation of muscle fibers by silencing Msc expression in the advanced phases of the differentiation process [66]. Interestingly, miR-378 is encoded within an intron of the peroxisome proliferator-activated receptor gamma coactivator 1-beta (Ppargc1β gene) that encodes for PGC-1β, a controller of energy metabolism. It has been suggested that miR-378 is co-expressed with PGC-1β, and it has been confirmed that it play a role in metabolism and interactions with target genes [66]. Gagan *et al*. (2011) showed that miR-378 is upregulated by MyoD during myogenic differentiation in immortalized mouse myoblast (C2C12) cells [72]. Chromatin immunoprecipitation (ChIP) and high throughput sequencing studies showed that MyoD is located close to miR-378 gene and leads to both transactivation and chromatin remodeling.

Transcriptional activity of MyoD is increased by miR-378 [73], partially by inhibiting MyoR that is an antagonist. The MyoR 3′ untranslated region (UTR) has a direct linking site for miR-378 and the presence of this linking site reduced the MyoR capacity to avoid the MyoD-driven transdifferentiation of fibroblasts notably.

### miRNA expression associated to muscle regeneration induced by physical activity

Exercise affects skeletal muscle and it was also recently shown it is capable to modify skeletal muscle miRNA expression [74, 75].

A group of young and adult males underwent for 12 weeks to resistance physical training separating the group into “low-responders” and “high-responders” based on each subject's modifications in lean body mass [76, 77]. In this study it has been analyzed if expression levels of the most expressed miRNAs varied among the two groups. It has been profiled twenty-one miRNAs, demonstrating that miRNA expression was not influenced in the vastus lateralis muscle in the high-responder group whereas the low-responder group showing an important modification in miR-451 and miR-378, with a downward tendency for miR-26a and miR-29a in the vastus lateralis muscle. miR-378 upregulation expression demonstrated an important association with lean body mass, leading the authors of this study to hypotesize that the expression of significant miR-378 levels were necessary for a lean body mass increase [76, 77]. *In vitro* data supported the idea that miR-378 contributes to myoblast differentiation by targeting MyoR, a negative controller of the myogenic transcription factor MyoD [77, 78]. Moreover, currently miR-378 has been demonstrated to regulate mitochondrial metabolism and bioenergetics via the modulation of PGC1-β, and its increased expression would likely be a normal condition as a consequence of endurance exercise [77, 79].

These data suggested that miRNAs play an important role in regulating the translation of key pathways responsible for skeletal muscle growth in response to RT in high and low responder individuals.

Ceccarelli *et al* (2017) showed that, 4 hours post-exercise, only a resistance exercise followed by cycling (a combination of anaerobic and aerobic exercise) caused an important boost in miR-23a-3p (~90%), miR-23b-3p (~39%), miR-133b (~80%), miR-181-5p (~50%), and miR-378-5p (~41%). A recent review reported gene expression regulations by exercise-related miRNAs [2, 80]. miRNA targets have been reported to be: 1) signaling transduction pathways controlled by calcium and activated protein kinase (AMP); 2) class IIa histone deacetylases; 3) muscle specific transcription factors (i.e. MyoD, MyoG); 4) mitochondrial targets mtTFA and FoxJ-3/MEF-2); 5) mitogen-activated protein kinases (MAPKs); 6) Run 1, Sox9, Pax3; 7) vascular endothelial growth factor (VEGF) and IGF-1 (Table 1) [2]. Taken together these findings evidenced a role for miRNAs in muscle metabolism modulation by physical training [2, 81].

myomiRNAs play an important role in the skeletal muscle adaptations to ET in both human health and diseases. ET can modify the levels of several miRNAs, e.g. miR-1, miR-16, miR-21, miR-26a, miR-29a, miR-126, miR-133a, miR-133b, and miR-206, miR-378, miR-451 and miR-494. Coordinated changes in the expression levels of these myomiRNAs contribute to skeletal muscle adaptations to acute and chronic ET. miRNAs can mediate the ET-induced changes in the skeletal muscle phenotype and exercise can rapidly and transiently regulate several miRNAs in the skeletal muscle.

Russell and colleagues (2013) have performed measurements in muscle biopsies of healthy men not practicing sports, after only one moderate-intensity endurance cycling or after chronic cycling ET [82]. They found a rise in muscle-augmented miRNAs, involving miR-1, -133a and 133-b, as well as miR-181a and in parallel the decrease of miR-9, -23a, -23b and -31; the latter miRNAs categories are increased in different muscle wasting diseases [82].

After short-term exercise of 10 days miR-1 was found upregulated, instead miR-29b and -31 were found downregulated [82]. Either loss- and gain-of-function experiments have shown that miR-208b and miR-499 play important roles in muscle fiber identity by activating slow and repressing fast myofiber gene program [82, 83]. Several other studies have shown that after 3 hours of one acute exercise miR-1, miR-133a, miR-133-b and miR-181a were all increased [10, 84, 85] whereas, miR-9, miR-23a, miR-23b and miR-31 decreased (Table 2). In another study, Nielsen and colleagues (2010) demonstrated that miR-1 and miR-133a expression levels in the vastus lateralis of healthy individuals were significantly upregulated after only one cycle ergometer activity at 65% of maximal power (Pmax). On the other hand, 12 weeks of ET on a cycle ergometer resulted in a decrease in the miR-1, miR-133a, miR-133b, and miR-206 [86–91]. Likewise, Keller *et al* (2011) showed that 6 weeks of cycling reduced the expression of miR-1 and miR-133 with miR-101 and miR-455 in human skeletal muscle [77, 92].

**Table 2:** Sport exercise affect skeletal muscle miRNA expression

miRNA upregulated	miRNA down regulated	Type of exercise	References
miR-1, miR-133a, miR-133b, miR181a	miR-9, miR-23a, miR-23b, miR-31	Acute exercise	Russell AP. *et al*., 2013.
	miR-1, miR-23a, miR-133a, miR-133b, miR-206	Acute resistance exercise, Chronic training, Chronic resistance exercise	Ringholm S. *et al*., 2011; Drummond MJ. *et al*., 2008, Nielsen S. *et al*., 2010, Mueller M. *et al*., 2011.
miR-1, miR-29b		Endurance	Russell AP. *et al*., 2013.
	miR-133a, miR-378, miR-486	Resistance exercise	Fyfe JJ. *et al*., 2016.
miR-136, miR-200c, miR-376, miR-377, miR-499b, miR-558	miR-28, miR-30d, miR-204, miR-330, miR-345, miR-375, miR-449c, miR-483, miR-509, miR-520a, miR-548, miR-628, miR-653, miR-670, miR-889, miR-1245a, miR-1270, miR-1280, miR-1322, miR-3180	Chronic resistance exercise	Ogasawara R. *et al*., 2016.
miR-451	miR-26a, miR-29a, miR-378	Resistance exercise	Davidsen PK. *et al*., 2011.

The list summarize which miRNA is up-or down-regulated in skeletal muscle after physical activity.

Data suggested that miRNA expression levels could modify following exercise status in order to control exercise adjustments [77].

Interestingly, miR-1 and miR-133, clustered on the same chromosomal loci, are transcribed together in a tissue specific manner during development and have distinct roles in the modulating skeletal muscle proliferation and differentiation [10]. Furthermore miR-1 and miR-133 are both implied in cardiac and skeletal-associated human disorders [93]. These results showed that these mature miRNAs, originated by the same miRNA polycistron and transcribed together, can perform different biological roles.

Other analysis showed that ET can modify the levels of several miRNAs expressed in skeletal muscle, such as miR-1, miR-16, miR-21, miR-26a, miR-29a and miR-126 [94, 95] (Table 2). In addition, changes has been reported in skeletal muscle miRNA expression such as miR-136, miR-200c, miR-376, miR-377, miR-499b and miR-558. These miRNAs were up-regulated after chronic resistance exercises (i.e. leg press, leg curl, etc.) while, several miRNAs, such as miR-28, miR-30d, miR-204 and miR-330 were on the contrary down-regulated [96] (Table 2). Taken together, the studies reported suggest that myomiRNAs play an important role in the skeletal muscle adaptations to ET in human health and that coordinated changes in the expression levels of these miRNAs contributed to skeletal muscle adaptations to acute and chronic ET.

### miRNA expression in skeletal muscle disorders

miRNAs are fundamental modulators of skeletal muscle health and it is of high interest their involvement in the onset and development of myopathies and chronic disorders related with muscle wasting and dysfunction [74].

Primary skeletal-muscle diseases include a cohort of pathologies, such as muscular dystrophies, inflammatory myopathies and congenital myopathies. Although every year the gene numbers implied in muscle diseases rise and histological pathology of disorder tissue is widely reported, the essential molecular mechanisms continue to be little described [97]. The most often inherited neuromuscular disease in adults is myotonic dystrophy type 1 (DM1) and in a study it has been demonstrated that miR-206, a regulator of muscle regeneration, was especially increased in patients affected by DM1 when compared to healthy individuals [98]. Furthermore miR-1 and miR-335 are elevated whereas miR-29b, miR-29c and miR-33 are reduced in patients affected by DM1, when compared to control individuals not presenting any pathological feature [99]. These miRNAs play roles in the regulation of muscle development [98]. Moreover, the cellular localization of miR-1, miR-133b and miR-206 seems to be scattered in DM1 muscle. Likewise, 11 miRNAs, involving the muscle enriched miRNA miR-208, are downregulated in the phosphatidylinositol-4,5-bisphosphate 3-kinase (PI3K)/AKT and in the transforming growth factor-β (TGF-β) signaling pathways of patient muscle samples affected by myotonic dystrophy type 2 (DM2) [100]. In fact, the downregulation of TGF-β cascade has been involved in both inherited and acquired unhealthy status influencing skeletal muscle, and Akt2 expression is highly enhanced during skeletal muscle cell differentiation and myocyte growth, indicating an Akt2 crucial function in myogenesis [100].

Some reports linked miRNAs with several muscle-related diseases, such as Duchenne muscular dystrophy (DMD), Becker muscular dystrophy, facioscapulohumeral muscular dystrophy, limb-girdle muscular dystrophies types 2A and 2B, Miyoshi myopathy, nemaline myopathy, polymyositis, dermatomyositis, and inclusion body myositis [54, 97]. miRNAs such as miR-1, miR-21, miR-33, miR-133 and miR-206 derived from patient serum affected by Duchenne muscular dystrophy showed that specific muscle-enriched miRNAs were widely upregulated in expression related with the development of the dystrophic disease [101]. The same findings were reported in dystrophic mdx mouse muscles (these spontaneous mdx mutant mice do not express dystrophin and may be useful for studying Duchenne muscular dystrophy, they are also known as DMD), which revealed that expression levels of miR-206 were widely enhanced in the muscle when correlated with normal mouse muscles [102], indeed, miR-206 levels are elevated in the diaphragm muscle of an animal mouse model of muscular dystrophy [54]. In addition, in a murine model of skeletal-muscle hypertrophy the expression levels of miR-1 and miR-133a were reduced [54]. In murine models, these downregulations could be overcome with therapeutic treatments, such as HDAC inhibition or recovery of nitric oxide (NO) signaling [103]. In DMD derived samples, miR-31 and miR-486 were recognized as regulators of muscle regeneration. In DMD myoblasts from humans, miR-31 silencing enhances dystrophin and therefore miR-31 regulation is suggested as a probable curative approach to ameliorate the DMD phenotype [104]. miR-486 expression was not modified in patient muscles affected by Becker muscular dystrophy who displays in part functional dystrophin protein [97]. miR-486 is suggested to play a significant regulatory role in the phosphatase and tensin homolog (PTEN)/Akt signaling pathway in dystrophin deficient and normal muscle [56]. miRNA expression profiling of the serum from the beagle-based canine X-linked muscular dystrophy in Japan (CXMDJ) model also demonstrated a downregulation of miR-1, miR-133a, and miR-206 [105]. Further analysis of serum obtained from DMD boys showed that besides three myomiRs (miR-1, miR-133a/b and miR-206) being enhanced in expression, two other muscle-enriched miRNAs, miR-208b and miR-499 raised [106]. miR-199a-5p is elevated in human DMD samples when correlated to the irrespective healthy subjects. It has been showed that miR-199a-5p turns off the expression of different members of the Wnt signaling pathway, a pathway that controls satellite cell sustenance and differentiation [107].

Overall miRNA expression-profiling analysis reported that a total of 185 miRNAs were downregulated in samples of pathological muscle tissue from ten muscle diseases (Duchenne muscular dystrophy, Becker muscular dystrophy, facioscapulohumeral muscular dystrophy, limb-girdle muscular dystrophies types 2A and 2B, Miyoshi myopathy, nemaline myopathy, polymyositis, dermatomyositis, and inclusion body myositis). Among those, five miRNAs (miR-146b, miR-221, miR-155, miR-214 and miR-222) were widely regulated by a post-transcriptional mechanism in almost all samples derived from all diseases that were examined [97, 108]. A direct genetic link has been associated to miRNA role in muscle hypertrophy [109, 110]. An aberration responsible for the extraordinary muscularity of Texel sheep has been mapped to a single G-to-A mutation into the 3′ UTR of the mRNA encoding myostatin, a component of the TGF-β family; myostatin role is to inhibit muscle growth. This alteration generates a linking site for miR-1 and miR-206, contributing to the translational inhibition of myostatin, which phenocopies the “muscle doubling” that results from the loss of myostatin in mice, cattle and humans [110–112].

It has been reported an increase in miR-23a in skeletal muscle of patients affected by amyotrophic lateral sclerosis (ALS) when correlated to healthy subjects [113]. *In vitro* it was determined that miR-23a controls peroxisome proliferator-activated receptor gamma coactivator 1-alpha (PGC-1α) negatively [113], that is a fundamental stimulator of mitochondrial biogenesis and function. It has therefore been hypothesized that curative repression of miR-23a could deliver PGC-1α function and improve ALS phenotype. Sixteen miRNAs such as miR-1, miR-28–5p, miR-19b, miR-100, miR-127–3p miR-135, miR-192 were downregulated in patients affected by laminopathies, a class of myopathies inducing alterations in the lamin A/C gene [114]. Pathway enrichment analysis in the predicted targets of these miRNAs revealed pathways involved in muscle repair, such as MAPK, TGF-β and Wnt signaling. miR-100, miR-192 and miR-135, were involved in C2C12 myoblast growth and differentiation. In children muscle affecting by dermatomyositis, it was observed that 33 miRNAs resulted elevated [97], miR-126 was significantly decreased in subjects in the first stage of pathology when related to control subjects [115] and it has been suggested to play a particular function in the first but not in the last stage of juvenile dermatomyositis by contributing to the expression of the vascular cell adhesion molecule 1 (VCAM-1), a protein usually demonstrated in the progression but not in the complete muscle fibers.

Facioscapulohumeral muscular dystrophy (FSHD) is due to genetic modifications including the long (q) arm of chromosome 4. This disorder derives from modifications in a region of DNA close to chromosome termination known as D4Z4. Hypermethylation of the D4Z4 region usually keeps a gene named DUX4 that is repressed in most adult cells and tissues. The DUX4 gene is placed in the segment of the D4Z4 region near to the final part of chromosome 4 [116].

The expression of the transcription factor DUX4 and its transcriptional regulation have been reported to play an important downregulation of miRNAs during FSHD disorder progression [117]. The upregulation of miRNA-411 in FSHD myoblasts has been described as a possible process for the interruption of myogenic differentiation through direct suppression of transcriptional repressor protein YAF2 and YY1 transcriptional role [118]. Full transcriptome study of miRNAs dysregulated in FSHD myoblasts and serum from FSHD patients reported an important enhance in expression of the muscle myomiRs (miR-1, miR-133a/b, miR-206) with important downregulation of different miRNAs [116]. Next-generation sequencing of FSHD myoblasts (RNA samples extracted from muscle cells) reported several additionally dysregulated miRNAs when compared with unaffected patient myoblasts, such as miR-1, miR-133a/b and miR-206 [119].

The downregulation of myogenic and non-myogenic signaling cascades that happen in the FSHD disorder status [120] is related to miRNA alterations and influence FSHD disease progression.

miRNAs are fundamental in the control of several gene networks and signaling cascades in muscle, therefore, they are significant regulators of skeletal muscle health and several miRNAs are downregulated in specific muscle disorders status [121]. Based on the results obtained in these studies, miRNAs such as, miR-1, miR-133a/b, miR-206 could be considered as key regulator in this kind of dystrophy [116].

miRNAs have been identified in most biofluids, such as serum and plasma and there is a rising interest in analyzing circulating miRNAs (c-miRNA) as biomarkers of normal or pathological mechanisms [122, 123]. Controlling the status of skeletal muscle has been a long-time concern and there is constant, ongoing research to find new blood markers of muscle damage [123]. Data published shows that miRNAs are consistent biomarkers of both acute and chronic muscle injury and may be a result of adaptation to physical activity [123]. Several applications could result in both research and medical field [123]. Physical activity physiology and sports science could take advantage from novel biomarkers able to analyze muscle conditions in response to exercise [123]. MyomiRs can also reflect muscle mass or muscle loss. Muscle wasting is a mechanism and muscle mass a symptom; since both can often determine morbidity or mortality, circulating myomiRs are hopefully novel diagnostic/prognostic instruments to be used in the medical field [123]. Different myomiRs have been identified in skeletal muscle as well as in cardiac muscle [123]. It has been reported that several muscle-specific miRNAs, including miR-1/206, miR-133 and miR-208 are fundamental for normal myoblast differentiation and growth and they have also been involved in several cardiac and skeletal muscular disorders suggesting that miRNA-based gene approaches could have a prospective for the cure of cardiac and skeletal muscle diseases [124].

## SKELETAL MUSCLE REGENERATION AND AGING

In our modern society aging is one of the important challenges. Advanced adult age is associated with changes in many physiologic systems. Of particular interest is the musculoskeletal system because it directly contributes to mobility and functional independence. Skeletal muscle mass and strength decline with age [125]. These changes are mostly due to a decrease in the quantity of muscle fibers and cellular and molecular changes that reduce the force-generation process [23].

In aging, bone mass and architecture are compromised and may result in fractures as well as tendons and ligaments undergo significant biochemical alterations that directly compromise their biomechanical function [126].

The age-related reduction in muscle repair efficiency contributes to the development of sarcopenia, one of the most important factors of disability in elderly people. During aging, several tissues undergo modifications in stem cell number and fuction, that impact tissue homeostasis. Specific extrinsic mediators from local and systemic environment are necessary for stem cell role. Aging of the stem cell either in local and systemic environment is related to stem cell death. Since the first evidence that muscle restore was under the control of soluble components present in serum, modifications in the content of the systemic environment has been the predominant model to identify impairments in skeletal muscle restore during aging [127].

In studies using old mice, muscle restore is blunted in wide part for satellite cell dysfunction [128, 129].

In other types of stem cells, such as hematopoietic stem cells, not only the role but also the quantity of satellite cells decrease with aging. In aged muscle, the quantity of stem cells could be reduced during the regeneration process [130]. It seems that there is a minimum number of muscle stem cells to effectively restore muscle mass and the quantity will be due to the fitness of the cells and the environmental support. With age, Notch pathway stimulation becomes defective for reduced delta ligand expression in myofibers and in satellite cells. This decrease in Notch stimulation is also expanded by excess of TGF-β/phospho-Smad (pSmad), leading to an aggregation of cyclin-dependent kinase (CDK) inhibitors in muscle stem cells, hence avoiding their regenerative responses [131, 132]. Parabiosis and all muscle grafting experiments revealed that processes regulating the regeneration ability of stem cells may be particularly due to modifications of the local environment during aging [130]. In elder population indeed, the efficacy of muscle regeneration decrease, promoting that the satellite cells function and their progeny may be altered. Satellite cells are not isolated but are rather encircled and affected by several extrinsic components that are in connection with the stem cell and the stem cell niche, autocrine and paracrine elements (both at rest and after injury) and with circulating elements that can modify their function. These elements probable modify during aging and induce both reversible and irreversible modifications to the satellite cells and on their proliferating progeny.

### Age-associated miRNA regulation in skeletal muscle

Sarcopenia, the age-related wasting of skeletal mass and role, enhances falls and fractures worsening the independency of life [133]. Analysis are ongoing with a larger sample size to determine if the age-related modifications in skeletal muscle are affected by miRNA aberrant expression and activity. Moreover, it will be significant with regard to the prospective of exercise levels and nutritional conditions, to consider all elements which can affect muscle miRNA levels, when comparing young and older individuals. It has been reported high levels of pri-miR-1-1, -1-2, -133a-1 and -133a-2, with no modification in pri-miR-206 in skeletal muscle biopsies taken from six aged (70 ± 2 years) subjects when related with six young (29 ± 2 years) men [88]. The potential of miRNAs to modulate aging in model organisms has recently attracted the interest of the molecular genetics community [134]. Numerous miRNAs, such as miR-71 in C. elegans and miR-17-92 in mammals, have been demonstrated to be particularly up- or down-regulated in aging [135, 136]. These advancements have given a further comprehension of specific elements that regulate aging signaling pathways in different species from C. elegans to humans. Interestingly, let-7 miRNA [137] is downregulated with age in C. elegans [135, 138]. Indeed, analysis in older individuals have determined that two miRNA levels from the let-7 family of miRNAs, let-7b and let-7e, are higher in skeletal muscle when correlated to young participants [88, 139]. The sequence of C. elegans (cel)-let-7 differs poorly from human (hsa)-let-7b and hsa-let-7e and this discrepancy could be enough to give various mRNA targets for these three miRNAs. In both C. elegans and humans the role of the let-7 family of miRNAs is similar. The primary function of the let-7 miRNAs seems to be anti-proliferative, as identified in human tumor cells [140–144] and in mouse neuronal stem cells [141]. The increase of let-7 miRNAs could be the reason for the damaged capacity to stimulate and induce the proliferation of satellite cells in the elderly skeletal muscle, hence leading to the mitigated skeletal muscle regenerative ability in the aged [145]. Therefore, bioinformatic studies detected cell cycle control and cell cycle progression and proliferation as the cellular mechanisms probable to be modulated in humans by the 2 let-7 miRNAs.

Atrophy of skeletal muscles can origin from either primary or secondary muscle diseases or from inactivity and/or aging in healthy subjects [146, 147].

Analysis made at bed rest on healthy human subjects, as well as animals atrophy models (mouse and rat), demonstrated a dysregulation of myomiRs, which not contribute to inhibition of pathways such as insulin signaling, TNF, TGF-β Smad2, and MAPK inducing progression of atrophy, insulin resistance, and metabolism and fiber type shift [87, 97, 147, 148].

To distinguish among miRNA modifications in “normal” and disorder-induced muscle atrophy, it is fundamental to correlate both mechanisms. In healthy subjects, skeletal muscle atrophy derives from continued immobility, aging, caloric limitation, physical passivity, or particular microgravity status, such as space flight and is represented by decreased muscle force, lower synthesis and higher protein degradation rate, protein carbonylation, shift in muscle fiber type from slow type I to fast type II, enhanced oxidative stress, development of insulin resistance, and intramuscular fat deposits [149]. It was demonstrated that age affects myomiR expression in an analysis correlating skeletal muscle biopsies of young and older healthy men [139]. In older men, let-7a/b/e/f and miR-25, miR-98, miR-195, and miR-1268 were increased, whereas miR-22, miR-24, miR-27a, miR-27b, miR-30d, miR-133a, miR-133b, miR-223, and miR-278 were dysregulated when compared to young participants [139]. There was no difference in the expression of miR-206 among these cohorts.

## CONCLUSIONS

The emergence of the miRNA field contributes to a sensational possibility to deepen the knowledge of molecular components which regulate skeletal muscle development, regeneration and aging. Moreover, miRNA biology also provides an avenue to dissect the mechanisms which may contribute to genetic and acquired muscle diseases and related complications. The impact of behavioural choices influencing physical activity has an important role in determing our longevity and quality of life. The observations that physical exercise may influence miRNA levels has important implications to understand how to maintain health throughout the lifespan, an issue of great relevance considering our aging and sedentary communities. A downregulation of different miRNAs occurs in myopathies, in chronic disorders associated to muscle loss as well as in aging. These studies suggested that skeletal muscle miRNAs play an important function in muscle adaptation or maladaptation to endurance and resistance exercise training. The recognition of miRNAs and their modulation following physical activity suggest that they could be helpful biomarkers of healthy status and might be amenable for future therapeutic intervention.
